# Menstrual hygiene management practices among primary school girls from a pastoralist community in Kenya: a cross sectional survey

**DOI:** 10.11604/pamj.2018.31.222.13521

**Published:** 2018-12-05

**Authors:** Eleen Korir, Florence Nafula Okwara, Gaudencia Okumbe

**Affiliations:** 1 Department of Community Health, School of Public Health, Kenyatta University, Nairobi, Kenya; 2 Department of Paediatrics and Child Health, School of Medicine, Kenyatta University, Nairobi, Kenya; 3 Department of Environmental Health, School of Public Health, Kenyatta University, Nairobi, Kenya

**Keywords:** Menstrual hygiene management, adolescents, rural pastoralist communities

## Abstract

**Introduction:**

pubescent girls from developing countries are confronted with diverse menstrual hygiene management (MHM) challenges, especially at school. Girls from rural pastoralist communities experience insurmountable MHM barriers. Inadequate coping strategies adopted result in sub-optimal school performance, absenteeism and physical problems. We conducted a study to assess MHM practices among primary school girls from a pastoralist community in Kenya.

**Methods:**

a cross sectional survey was done among primary school girls in Kajiado County, Kenya. Accent was sought. We administered structured questionnaires which sought information on socio-demographics, knowledge, perceptions and practices.

**Results:**

we enrolled 320 girls; with mean age of 14.9 years. Their parents were mostly (69.4%) self-employed pastoralists. Good menstruation knowledge was observed in 51.6%, while 45.5% reported diverse perceptions about menstruation. Majority, (80.9%) used sanitary towels as absorbents, but 40.3% delayed changing by > 6 hours. Poor MHM practices were documented in 28.8% and 32.2% kept the issue secret. Factors associated with poor MHM practices on univariable analysis were age (p=0.016), religion (p=0.037), non-discussions (p=0.001), lack of sanitary pads (p<0.0001), lack of latrine privacy (p=0.031), lack of water (p=0.001) and teasing by boys (p=0.016). On logistic regression, factors that independently influenced MHM practices were inadequate latrine privacy (p=0.031) and fear of teasing by boys (p=0.016).

**Conclusion:**

a third of pubescent pastoralist girls had poor MHM practices largely determined by inadequate latrine privacy and fear of teasing by boys.

## Introduction

Pubescent girls from developing countries, especially those from rural settings face diverse “Menstrual Hygiene Management” (MHM) challenges [1-4]. Many rural schools are not attuned girls' menstruating needs. Girls from pastoralist communities face insurmountable MHM barriers [5, 6]. MHM is defined as use of clean menstrual material to absorb or collect blood that can be changed in privacy as often as necessary for the duration of menstruation, using soap and water for washing the body as required and having facilities to dispose of used menstrual management materials [7]. Unfortunately, many rural schools have inadequate water, sanitation and hygiene facilities (WASH) for girls [2-6, 8-10]. According to the Kenya Demographic Health Survey (KDHS) 2014, school latrine coverage stood at 49.5% [11]. Many schools lack clean and private changing rooms [6, 8]. Many girls cannot access or afford appropriate absorbent materials and often resort to crude methods [5, 6, 10]. Besides, menstruation is surrounded by divergent religious beliefs and cultural perceptions that impact on MHM practices [5, 6, 10]. It is considered a taboo by various communities and even by the teachers; hence they do not provide information and guidance on the meanings and management of menses [4]. Unable to cope with this physiologic process and to avoid suffering shame, girls adopt diverse coping strategies that vary across regions, based on personal preferences, resources available, knowledge and cultural beliefs [4]. This affects their rights, social and mental well-being, resulting in sub-optimal school performance, school absenteeism, and drop outs [12-14]. On average, adolescent girls miss at least 6 weeks of school per year due to menses [13, 15]. Further to, many suffer diverse reproductive health problems, especially urogenital infections arising from unsafe unhygienic practices [6, 16]. This has worsened existing gender disparities observed across regions on access, retention, transition and achievements on education [13]. To mitigate its effects, in 2009, the Government of Kenya increased the budgetary allocation to schools to improve WASH facilities and to provide subsidized sanitary pads for needy girls [17-19]. We conducted a study to assess the MHM practices and challenges among primary school girls from a rural pastoralist community in Kenya.

## Methods

**Design, setting and study population:** we conducted a school-based cross-sectional survey in Mashuru Sub-county, Kajiado County, Kenya. Ten out of 47 primary schools in the county were randomly sampled. The schools were stratified by classes, and only girls in classes 4-8 were included. Boys and girls that had not attained menarche were requested to leave the classrooms. Systematic random sampling was done, using a sampling frame generated from class lists. Girls absent on the interview day and those non-residents in the area in the preceding 3 months were excluded. Written accent was sought from the girls. For those below 18 years, parental consent was sought giving the girl a form to be signed by the parent of guardian. Subject enrollment started in December 2014 to February 2015. We administered a structured questionnaire that sought information on socio-demographics, economic, knowledge, perceptions and MHM practices. Knowledge sought covered causation and origins of menstrual blood. The Likert scale was used to categorize knowledge into good (≥50%), average (49-30%) and poor (<30%) based on correct responses. MHM practices were determined based 4 actions; type of absorbent materials used, frequency of changes, appropriate disposal and regular hand washing after toilet use. For those using reusable absorbents, the storage place of absorbents was considered instead of disposal. Each appropriate action was assigned a score of 2, and those with total score ≤4 out of 8 were considered to have poor MHM practices. In-depth interviews with school administrators provided qualitative data. An observation checklist was used to determine status of WASH facilities in the schools.

**Data management and analysis:** consecutive eligible girls were enrolled, until desired sample size of 320 girls was attained. Oversight and safety monitoring was done by the Kenyatta University Ethics Review Committee (KU-ERC). Data collected was coded, entered and analyzed using Statistical software; SPSS version 21. The characteristics of respondents are presented as frequencies, percentages, means and ratios. Comparisons were made of dependent variables observed in those with poor versus those with good MHM practices. Cross tabulations were done and associations established by chi -squares. Inferential statistics (p-value, confidence intervals) were used to establish factors associated with poor MHM practices. Statistical significance level was fixed at p<0.05. For advanced statistics, factors showing significant associations on invariable analysis were entered into a logistic regression model, to establish those with an independent relationship with optimal MHM practices.

## Results

**Subjects' enrollment and participants:** out of a total of 648 eligible girls, 247 were pre-menarche and 45 were absent from school on day of interview. We sampled 356 girls, but 24 girls declined participation, while 12 were excluded based of non-residency, hence 320 girls were enrolled in the study.

**Socio-demographic characteristics of respondents:** the mean age of the girls was 14.9 years (SD=1.8), while mean age at menarche was 13.0 years (SD=2.1). Majority (69.4%) of their parents were self-employed pastoralists. Most girls (58.7%) were in classes 7 and 8. Most, 90.9% were of Christian-protestant faith, though 3.1% were Muslims and 5.9% belonged to traditional religious sects.

**Knowledge level on menstruation among respondents:** overall, 51.6% had good menstruation knowledge, but regarding causation, only 46.4% knew that it was a physiologic process, and 26.6% thought it was a curse. On origin of menstrual blood, 48.4% reported it was from the uterus. Most (72.8) girls were aware about menstruation at the time of menarche. Regarding the source of menstrual information, majority (69.1%) learnt about it from a teacher, and only 19.4% from a parent. Knowledge assessment details are provided in Table 1.

**Table 1:** level of knowledge of respondents on menstruation

Characteristic	Category	Number (N= 320)	Percent (%)
Overall knowledge	Good	165	51.6
	Average	75	23.4
	Poor	80	25.0
Knowledge on causation	Physiologic process	148	46.4
	Sin	25	7.8
	Curse	85	26.6
	Disease	65	20.3
	I don’t know	1	0.03
Knowledge on source of blood	Uterus	154	48.4
	I don’t know	165	51.6
Source of information on menstruation*	Teacher	221	69.1
	Television/television	18	5.6
	Newspapers/magazines	14	4.3
	Health talks	15	17.2
	Parents	62	19.4
	Others	2	8.1

* Respondents had more than one response

**Cultural perceptions on menstruation:** most participants (96.8%) were from the Maasai community, which is largely nomadic. There were varied perceptions on menstruation reported in 45.5% of the girls. Stigma was reported by 37.8%, shamefulness (44.7%), fear (23.3%), and 17.5% felt it was a private matter. From in-depth interviews, because menstruation was considered a taboo in this community, discussions on the matter in public was prohibited. This heightened stigma and social silence. Further, Muslim and traditional religious groups considered menstruating girls unclean and prohibited their participation in any social forums, including school, which left girls ostracized and alienated. The Maasai culture prohibited girls from sharing latrines with boys, hence girls preferred to use nearby bushes around school. Because of their nomadic culture, most homesteads and schools did not construct proper latrines.

**Menstrual hygiene management practices of respondents:** majority, (85.9%) used disposable absorbents, mainly sanitary towels (80.6%), which were obtained from school in 57.5% cases. From in-depth interviews, it was reported that sanitary towels were supplied to schools once per term and rations were dependent on the population of girls in the school. Supplies were limited and would get depleted before end of term; hence girls had to seek alternative sources. At least 40.3% girls delayed changing by >6 hours, and this emanated from inadequate absorbents (68%) and long distance to private changing areas (32%). Most (81.2%) disposed used pads in pit latrines, while 11.6% disposed in dust bins. Of those using reusable absorbents (14.1%), half used pieces of cloth, and most (72.7%) kept them in hidden and unclean places. Only 54.7% girls reported to always wash hands after visiting the toilet, as sometimes there was no water at school. Besides, only 47.5% had daily bathing during menstruation days. We observed poor MHM practices in 28.8% girls and 32.2% kept the matter secret. The first person informed on onset of menstruation was the mothers (45.0%). Among challenges were expressed by the girls, included inadequate sanitary pads (39.1%), menstrual pains (33.8%), 39% reported that they had suffered disease of the skin or the reproductive system due to poor practices. Details of the MHM practices and challenges are presented in Table 2.

**Table 2:** menstrual hygiene management practices and challenges

Characteristic	Category	Number (N= 320)	Percent (%)
Absorbent materials used	Sanitary pads	258	80.6
	Toilet paper	6	1.9
	Pieces of cloth	24	7.5
	Tampons	11	3.4
	Menstrual cup	20	6.3
	Others (Sponges)	1	0.003
Source of absorbent material	School	182	57.5
	Shop	80	25.0
	Home made	50	15.6
	Others	7	2.2
Frequency of changes of absorbents (hours)	3-6	194	60.6
	> 6-12	80	25.8
	>12	46	14.5
Bathing during menses	Always	152	47.5
	Sometimes	122	38.1
	Rarely	46	14.4
Hand washing after visiting the toilet	Always	175	54.7
	Sometimes	107	33.4
	Rarely	38	11.9
Disposal of disposable absorbent material	Pit latrine	224	81.2
	Dust bins	32	11.6
	Burn	7	2.5
	Burry in the ground	10	3.6
	Other	3	1.1
Discussions with others	Open discussion	18	5.6
	Selective discussion	198	61.8
	Never discussed	104	32.5
First person informed of onset of menstruation	Teacher	73	22.8
	Friend	89	27.8
	Mother	144	45.0
	Relative	14	4.4
Challenges experienced*	Inadequate absorbents	125	39.1
	Menstruation pain	108	33.8
	Lack of adequate water	48	15.0
	Lack of privacy	63	19.7
	Teasing by boys	17	5.3
	Skin/urogenital infections	117	39.0
	Others (insecurity, foul smell, fear, shyness, leakage, dislodgement)	19	5.9

* Respondents had more than one response

**School factors influencing MHM practices:** only 39.1% had received absorbents from school in the preceding month. Majority, (77.2%) reported inadequate clean water at school, 64.6% observed that the latrines were inadequate and 80.3% decried inadequate latrine privacy. Several girls (13.1%) reported that they had been laughed at or teased by boys, whenever they stained their clothes, which left them embarrassed and humiliated. There were some girls (5.8%), who wished that boys were educated on matters menstruation, so as to understand them better. Findings of the observation checklist showed that most (56.4%) schools had fewer latrines than recommended for the number of pupils registered. Various schools (65.0%), lacked privacy of latrines; reasons for this being that latrines were shared by both boys and girls in 10% , latrines lacked doors and/or locks in 24%, and some (31%) had informal non-secluded structures that did not offer privacy. Most (92%) schools did not have separate changing rooms for girls, and 98.6% lacked any sanitary disposal bins. At least 28% of schools lacked water for hand washing and 39% had unclean toilets. All sampled schools had mixed boys and girls, and this restricted open discussion.

**Determinants of menstrual hygiene management practices:** on univariable analysis, socio-demographic factors associated with poor MHM practices were age (p=0.016) and religion (p=0.037). Poor overall knowledge (p=0.001) and non- discussions of menstruation (p=0.001) were significantly associated with poor MHM practices. School factors found to be significantly associated with poor MHM practices included lack of provision of pads (p<0.001), lack of water (p=<0.001), lack of latrine privacy (p=0.025), and teasing by boys (p=0.016) (Table 3). Variables significantly associated with poor MHM practices on univariable analysis were included in the multiple logistic model to identify which ones had an independent relationship with MHM. The strongest independent determinants of poor MHM practices were, lack of privacy of latrines (p= 0.031) and teasing by boys (p=0.016).

**Table 3:** table of determinants of MHM practices

Characteristic		Category	MHM state (%)		X^2^ (df)	OR( 95% CI)	P value
			Good	Poor			
Socio - demographic determinants	Age (years)	**Reference age = Age 20 - 24 years**					
		10 - 14	37.2	13.8	2.197 (2)	0.246(0.094 - 0.643)	0.016
		15 - 19	29.1	13.8		0.315(0.120 - 0.827)	
		20 - 24	2.5	3.8			
	Occupation	**Reference = Employed**					
		Unemployed	47.3	21.9	0.181 (1)	1.12(0.67 - 1.87)	0.671
		Employed	21.6	9.1			
	Religion	**Reference = Traditional religion**					
		Christian	63.8	26.3	7.175 (2)	0.29 (0.11 - 0.77)	0.037
		Muslim	2.5	1.6		0.45 (0.11 - 1.92)	
		Traditional	2.5	3.4			
Knowledge determinants	Overall knowledge level	**Reference = good knowledge level**					
		Low	2.2	21.9	168.03 (2)	74.00(29.89 - 183.19)	<0.001
		Average	20.3	3.1			
Cultural perceptions	Open discussion of the matter	**Reference = No free discussion**					
		Yes	51.3	16.6	14.62 (1)	0.385(0.23 - 0.63)	<0.001
		No	17.5	14.7			
	Shamefulness	**Reference = Yes on shamefulness**					
		Yes	12.4	7.4	6.42 (1)	3.92(1.65 - 5.69)	0.056
		No	56.4	23.8			
	Stigma	**Reference = high stigma level**					
		High	31.3	13.7	12.5 (1)	4.92(1.92 - 7.49)	0.070
		low	45.4	9.6			
School factors	Provision of pads	**Reference = No sanitary pads provided**					
		Yes	22.8	19.7	23.08 (1)	3.43(2.054 - 5.751)	<0.001
		No	45.9	11.6			
	Latrine privacy	**Reference = No privacy provided**					
		Yes	12.0	9.0	5.145 (1)	1.93(1.09 - 3.43)	0.025
		No	56.9	22.1			
	Lack of water	**Reference = No water available**					
		Yes	7.8	8.5	11.67 (1)	2.920(1.55 - 5.49)	<0.001
		No	61.0	22.7			
	Teasing by boys	**Reference = No teasing**					
		Yes	2.4	3.4	6.42 (1)	3.415(1.26 - 9.28)	0.016
		No	66.4	27.8			

## Discussion

**Statement of principal findings:** poor MHM practices were documented in 28.8% girls. Most (80.6%) used sanitary towels that were provided by the school (57.5%), but supplies were inconsistent as observed in the preceding month, where only 39.1% had received supply. Many (40.3%) girls delayed changing absorbents by >6 hours. Of those using reusable absorbents (14.1%) and 72.7% of these stored them in unclean hidden places. On univariable analysis, poor knowledge level, non-discussion, lack of pads supply, lack of water and lack of latrines were associated with poor practices. Lack of latrine privacy and teasing by boys stood out as independent determinants of poor MHM practices.

**Strengths and weaknesses of the study:** this was a school-based study that focused on marginalized girls from a pastoralist community, who experience adverse climatic conditions, resource limitations and cultural barriers. Few studies have looked at MHM practices among pastoralist girls [9]. Progressive reduction in age of menarche has been observed in recent years, but many primary schools and communities remain unprepared for this reality [20]. School environments are often not conducive to MHM. This study focused on primary school girls. Various ongoing support initiatives have provided subsidized sanitary towels to disadvantaged girls, to keep them in school, hence need for re-evaluation of MHM practices. A selection bias may have arisen from exclusion of non-accenting girls and those absent on interview day, as menstrual problems may have been underlying. The cross-sectional design adopted may have masked any seasonal variations. Self-reports we used to determine practices, but this may not have been absolutely accurate, given the secrecy surrounding the subject.

**Strengths and weaknesses in relation to other studies:** previous studies have focused on types of absorbents used, as key barriers to optimal MHM practices [5, 8, 20, 21]. We focused on knowledge, cultural and school-based determinants of MHM practices; hence affordability and feasibility issues were not assessed. Various studies have addressed community-based approaches [1, 21, 22], but school environments further complicate the situation [8, 14, 23-26]. Others have focused on secondary level girls [4, 20, 27, 28], whose level of knowledge may be superior. Many studies collected qualitative rather than quantitative data. Various studies on MHM centered on urban schools [12, 29-32], whose dynamics differ from those in rural schools [2, 5, 10, 27].

Discussion of important differences in results: we documented a lower prevalence of poor MHM practices in these girls compared to the National prevalence in Kenya of 35.9% [11]. Lack of sanitary pads was not independently associated with poor MHM practices. This unexpected finding could be explained supplies support initiatives of subsidized towels in this community. Contrary findings have been reported in various studies where lack of sanitary towels was a key contributor to poor MHM, as girls used clothes and rags. This was observed in South Asia [9], in rural India where only 11.3% used sanitary pads [21], in Tanzania where 50% used sanitary pads [8], and in Ethiopia where only 35% were using sanitary pads [23]. Questions of sustainability of supplies may need to be addressed, by embracing local solutions. Many (40.3%) girls delayed changing absorbents, either due to limited absorbents and/or lack of private changing places, which meant that changing was delayed until some privacy was guaranteed. This factor overshadowed the benefit of provision of sanitary towels, as lack of latrine privacy was an independent predictor of poor MHM practices. Similar observations were made in South Sudan, where 56.6% reported lack of private changing rooms [8]. In study done in Western Kenya, girls experienced challenges in school due to inadequate privacy and sanitary facilities [2]. Crude storage methods were reported in girls using re-usable absorbents. Similarly, in India, girls recycled rugs and cleaned then without soap, or with unclean water. Most dried them indoors rather than in the sun, due to social restrictions which predisposed to reproductive tract infections [21]. In South Asia, girls recycled the same absorbent for several months to years [33]. Recycled absorbents should be made of materials that are low cost, easy to clean and are quick to dry, in order to prevent genital infection [25]. Knowledge on causation and origin of menstrual blood was low, but overall knowledge level did not independently influence poor MHM practices. Other African studies documented lack of scientific knowledge of menstruation and puberty among school girls [26, 30, 34-36].

In South Sudan, 62.9% considered menstruation as a disease [33]. In Kenya, very few girls were able to describe menstruation in biologic terms [29]. From the Kenyan National data, level of knowledge did not influence MHM practices [11]. However, some studies observed contrary findings [8, 30, 37]. Most girls learnt about menstruation from teachers, but not from their mothers. There has been a breakdown of traditional family methods of conveying maturation instruction (through grandmothers and aunties). Mothers often shied away from discussing this matter with their children, because of their own deficiencies in facts and good practices, instead passing on cultural taboos and restrictions, rather than physiologic or practical MHM facts [2, 22, 38, 39]. Even teachers avoided discussions on menstruation, mainly due to local taboos or due to their own inadequacies on the subject [33, 40], while some focused on the physiology, but not the practical management [23, 38, 41]. On the contrary higher knowledge rate (90.7%) was reported in Ethiopia, which was attributed to media campaigns and improved access to reproductive health information [23]. Alternative and innovative channels of sharing accurate menstruation information needs to be adopted for pre-pubescent girls to raise awareness on MHM. Non-discussion of menstruation was reported in 32.2% of the girls and was associated with poor MHM practices. Many cultures consider women unclean during menstruation, leading to social alienation and seclusion, and hence the silence witnessed on the matter [39]. Similar observations of non-discussions among family members were made in other Kenyan studies [4, 22, 27]. In India, society discouraged open discussion due to shamefulness and fear, which left girls withdrawn, anxious, and not fully involved in learning [41, 42]. One Sub-Saharan Africa study reported contrary findings [10]. Cultural restrictions are more commonly transmitted to the younger girls in their communities than knowledge, which poses barriers to adopting new practices. Some African cultures prohibit boys from sharing same latrines with girls, and menstruating girls were ostracized [43]. In India, cultural restrictions of washing and bathing were shown to influence MHM practices, despite good knowledge [41]. With observed declining age at menarche [42], both parents and girls are caught unprepared. Menstrual education needs to be taught earlier in the school curricular. Despite low literacy levels in this community, parent occupation did not influence MHM practices. Similar observations were reported in India, where education level and employment of the mothers were not associated with good MHM practices [44]. However, from a study done in Nigeria [45] and from another study in Rajasthan, father's occupation was a predictor of safe MHM practices [46].

UNICEF (United Nations Children's International Emergency fund) and the Kenya School Health Policy recommend that schools have sufficient latrine; at least 1 latrine to every 25 girls, and 1 latrine to 30 boys [19, 47]. Inadequate WASH facilities have been documented rural schools in the region [2, 6, 11], including this study. Similar observations were made in Ethiopia, where lack of water was as a determinant of poor MHM in schools [12, 19]. In our study, lack of latrine privacy was an independent contributor to poor MHM practices. Similar observations were made in a study from Sudan [8]. Inadequate school support leaves many girls preferring to be absent from school during menstruation [4, 39]. In study done in Western Kenya, lack of resources for menstruation led to disengagement from school and stigma [2]. In another Kenyan study, done in Nairobi (Urban) and Garisa (rural) schools observed that 86% and 53% respectfully, of girls missed ≥ 1 day of school every 2 months [24]. Similar findings were observed Ethiopia, where 40% girls missed school because of stress related to menstruation [22]. The girls were constantly alert for leakages, foul smell and discomforts and had poor concentration in class, which contributed to teasing by boys [4]. Teasing humiliates girls by causing stress and embarrassment, stripes their dignity, leaving them withdrawn and lowering their self esteem. In our study, teasing by boys was independently associated with poor MHM practices. In our settings, men/boys often lack facts on menstruation, hence cannot support the women/girls around them. It was suggested by 5.8% of the girls that educating boys on the matter, would help them cope better during such times. This concurs with findings made from a study done in South Sudan, where 43.8% girls reported that they were afraid of being made fun of by boys [8]. Likewise, in a Tanzanian study, many respondents suggested need to educate boys on menstruation [2, 46]. Adolescence is a crucial developmental stage characterized by heightened self-awareness and actualization, and this could be totally deranged by menstrual accidents, leading to reduced self confidence and emotional wrecks.

**Meaning of the study:** schools MHM tool kit, recommends that latrine cubicles are in a safe location, clean, covered by a screen, and have water supply, soap and a covered disposal bin for used absorbents. The facilities should be well maintained and gender-segregated for better MHM [47]. Unfortunately, needs of menstruating girls remain unmet. Other considerations for design and construction of School WASH facilities include; latrine privacy, and alignment to local cultural and religious beliefs, in order address physical, emotional and psychosocial expectations. There is need for a holistic school MHM package aimed at providing pre-pubescent girls with factual accurate scientific information [4] and teach appropriate MHM practices, and promote sexuality education from an early age.

**Unanswered questions and future research:** this study did not asses the contribution of menstrual problems to school absenteeism and performance. Future research should explore how to improve the role of mothers and teachers in MHM training for girls and how to increase awareness and engagement of men/boys to support good MHM practices.

## Conclusion

One third of pubescent school girls from the pastoralist community in Kajiado County, Kenya have sub-optimal MHM practices. The main contributors of this are lack of latrine privacy and fear of teasing by boys. We recommend investment in provision of private WASH facilities that are culturally acceptable and need to promote sexuality education on menstruation targeting both girls and boys.

### What is known about this topic

- Many pubescent girls have inadequate knowledge of menstruation;
- Lack of sanitary pads is a key barrier to optimal MHM practices;
- Secrecy on menstruation matters is common in many cultures.

### What this study adds

- Cultural and religious perceptions overshadow knowledge in determining MHM practices and should be integrated in any interventions;
- Inadequate and poorly designed school WASH facilities, which lack privacy are key barriers to optimal MHM practices;
- Pre-menarcheal health and sexuality education should target both girls and boys.

## Competing interests

The authors declare no competing interests.
